# Enhanced Tumor Accumulation of Low-Molecular-Weight Hyaluronic Acid/Chitosan Nanocomplexes for Photothermal Therapy

**DOI:** 10.3390/pharmaceutics15020613

**Published:** 2023-02-11

**Authors:** Gayoung Jo, Eun Jeong Kim, Hoon Hyun

**Affiliations:** 1 Department of Biomedical Sciences, Chonnam National University Medical School, Hwasun 58128, Republic of Korea; 2 BioMedical Sciences Graduate Program (BMSGP), Chonnam National University, Hwasun 58128, Republic of Korea

**Keywords:** photothermal therapy, near-infrared fluorescence imaging, nanocomplexes, hyaluronic acid, chitosan

## Abstract

Targeted phototheranostic nanosystems involving both cancer-specific near-infrared (NIR) fluorescence imaging and NIR light-induced phototherapy have shown great potential to improve cancer detection and treatment. In this study, a bifunctional nanocomplex based on low-molecular-weight hyaluronic acid (LMW-HA) and chitosan oligosaccharide lactate (COL) conjugating a zwitterionic NIR dye (ZW800-1) was rationally designed and prepared, and it was simultaneously used to enhance tumor accumulation and photothermal therapy (PTT). When HA-COL-ZW nanocomplexes were intravenously injected into mice bearing NCI-H460 tumors, HA-COL-ZW revealed increased tumor accumulation with prolonged tumor retention. Moreover, the ZW800-1 incorporated in HA-COL-ZW nanocomplexes showed excellent capability to convert NIR light into heat energy at the tumor site, acting as a PTT agent. Therefore, the targeted phototherapeutic HA-COL-ZW nanocomplex is a biocompatible and effective photothermal nanoagent, which could be a good candidate for future clinical use.

## 1. Introduction

In recent years, photothermal therapy (PTT) has been widely utilized as a noninvasive and selective phototherapeutic strategy for the treatment of various types of tumors by causing damage to cancer cells through the photothermal process of light-to-heat conversion [1,2]. Particularly, nanomaterial-based PTT has been recognized as one of the most promising therapeutic modalities and techniques for laser-induced tumor ablation, providing the advantages of high sensitivity and specificity [3]. To achieve the improved phototherapeutic effect with PTT, nanomaterials with photothermal effects should possess good biocompatibility and high tumor targetability. Although many different types of nanomaterials (e.g., synthetic-polymer-, noble-metal-, and carbon-based nanomaterials) have been developed and used as PTT agents, owing to their strong light absorption, high photothermal properties, and excellent photostability, most of them are not approved yet for clinical applications because of their poor biocompatibility and potential long-term toxicity [4,5].

To overcome the biosafety issues, natural polysaccharide-based nanomaterials, such as starch, cellulose, chitosan, pectin, alginate, and dextran, have been extensively exploited as ideal carriers for biomedical and pharmaceutical applications, because of their good safety, stability, hydrophilicity, biocompatibility, biodegradability, and chemical functionality [6,7,8]. Among them, chitosan oligosaccharide lactate (COL), which is the depolymerized product of chitosan with a lower degree of polymerization (≈30) and an average molecular weight (MW ≈ 5000 Da), has a higher water solubility and lower viscosity compared to chitosan, making it a more suitable candidate for pharmaceutical and biomedical applications [9,10]. Because of its good physicochemical properties, COL has been suggested as a nanocarrier for imaging agents and anticancer drugs owing to its efficient intracellular and site-specific delivery [11,12,13]. Previously, our group reported a tumor-targetable PTT agent (COL-ZW) by combining COL with ZW800-1 as a highly water-soluble zwitterionic NIR dye to improve tumor-targeted delivery of the heptamethine cyanine dye for use in fluorescence-guided photothermal cancer treatment [14]. Additionally, natural polysaccharide hyaluronic acid (HA) is well known to bind to CD44, a non-kinase transmembrane glycoprotein, extensively overexpressed in various cancer types, including prostate cancer, triple-negative breast cancer, lung cancer, pancreatic cancer, and brain tumors [15,16]. In this regard, HA has been widely utilized for potential therapeutic targets and prognostic markers for cancer therapy [17,18]. Thus, structure-inherent tumor-targeted HA has promising potential for active targeted delivery systems. Specifically, HA plays various important roles in the body, depending on the molecular weight of HA, typically ranging from 50 kDa to 20,000 kDa. In previous studies of low-molecular-weight HA (LMW-HA), it is noteworthy that the LMW-HA, lower than 6 kDa, would enhance tumor angiogenesis, whereas the LMW-HA with higher than 6 kDa would inhibit angiogenesis [19,20].

Importantly, it is reported that the cationic surface of chitosan-based nanoparticles significantly reduces their circulation time and bioavailability upon exposure to a biological environment [21,22,23], which is consistent with our previous study by using COL-ZW as a tumor-targeted PTT agent [14]. More importantly, it is also known that cationic chitosan-based nanoparticles can be decorated with anionic polysaccharides such as HA to decrease the rate of protein adsorption and macrophage uptake during systemic circulation [24,25]. In this study, we prepared nanocomplexes composed of COL-ZW and LMW-HA as biocompatible carriers for tumor-targeted delivery of PTT agents. The tumor accumulation of COL-ZW having short retention time in tumor could be significantly enhanced and prolonged by forming a nanocomplex with LMW-HA, thereby resulting in achieving excellent phototherapeutic efficacy. Therefore, the HA-COL-ZW nanocomplex has great potential as a biocompatible phototheranostic agent for tumor-targeted imaging and effective photothermal cancer treatment.

## 2. Materials and Methods

### 2.1. Preparation of HA-ZW, COL-ZW, and HA-COL-ZW

LMW-HA (average MW ≈ 8000–15,000 Da) and COL (average MW ≈ 5000 Da, >90% deacetylated form) were purchased from Sigma-Aldrich (St. Louis, MO, USA) and used without further purification. The amine-functionalized zwitterionic NIR dye (ZW800-AM) and carboxyl-functionalized zwitterionic NIR dye (ZW800-1) were prepared as described previously [26,27,28,29]. For HA-ZW, ZW800-AM (0.8 mg, 0.87 μmol) was conjugated to LMW-HA (10 mg, 0.87 μmol) in the presence of 4-(4,6-dimethoxy-1,3,5-triazin-2-yl)-4-methylmorpholinium chloride (DMT-MM; 0.5 mg, 1.8 μmol) in distilled water (DW, 1 mL) at ambient temperature for 12 h. For COL-ZW, ZW800-1 (1.9 mg, 2 μmol) was used to conjugate with COL (10 mg, 2 μmol) in the presence of DMT-MM (0.8 mg, 3 μmol) in DW (1 mL) at ambient temperature for 12 h. The crude mixtures of HA-ZW and COL-ZW were dialyzed against DW using a dialysis membrane tube (MWCO 3.5 kDa) to eliminate free dyes and coupling agents, respectively. Finally, nanocomplexes of the desired LMW-HA/COL-ZW molar ratio (1/1) were formed spontaneously upon mixture of COL-ZW with LMW-HA solution under magnetic stirring, owing to the strong electrostatic interaction between the cationic amino groups of COL-ZW and the anionic carboxyl groups of HA. The resulting nanocomplex suspension was kept under stirring for 10 min to ensure a complete system formation. Size distributions and zeta potentials of HA, COL, and HA-COL in DW were measured by dynamic light scattering (DLS; Nano ZS Zetasizer, Malvern Instruments Ltd., Malvern, UK).

### 2.2. Optical Property Measurement

HA-COL-ZW dispersed in phosphate-buffered saline (PBS, pH 7.4) was used to determine the optical property. The absorbance of HA-COL-ZW was detected using a fiber-optic UV-Vis-NIR (200–1025 nm) spectrometer (Ocean Optics, Dunedin, FL, USA). Further, the fluorescence emission spectrum of HA-COL-ZW in a wavelength range of 750–900 nm excited at 710 nm was confirmed by a SPARK^®^ 10M microplate reader (Tecan, Männedorf, Switzerland).

### 2.3. In Vitro Cancer Cell Binding and NIR Fluorescence Microscopy

NCI-H460 cancer cells were cultured in RPMI 1640 medium supplemented with 10% fetal bovine serum (FBS; Gibco BRL, Paisley, UK) and an antibiotic/antimycotic solution (Welgene, Daegu, South Korea) using 24-well plates. The cancer cells were incubated in a humidified atmosphere with 5% CO_2_ at 37 °C. The NCI-H460 cells at ~50% confluency covering half of the surface area were washed with PBS. Subsequently, the 2 μM concentrations of HA-ZW, COL-ZW, and HA-COL-ZW were, respectively, placed into each plate. The plates were stored in a CO_2_ incubator for 24 h. The NIR fluorescence imaging of each sample was conducted under a Nikon Eclipse Ti-U inverted microscope system (Nikon, Seoul, South Korea) after the washing step with PBS.

### 2.4. NCI-H460 Xenograft Mouse Model

Athymic nude mice (6 weeks old, the average adult weight is 25 g) were purchased from OrientBio (Gwangju, South Korea). The animal experiment protocol was approved by Chonnam National University Animal Research Committee (CNU IACUC-H-2020-19). The cultured NCI-H460 cancer cells were suspended in 100 μL of PBS to inoculate subcutaneously into the right flank of each mouse (1 × 10^6^ cells per mouse). At 8 and 10 days post-inoculation, the tumors of 1 cm or less in diameter were prepared to test the tumor-targeting capacity of the samples, as well as the PTT application. The mice injected intravenously with samples were euthanized for NIR fluorescence imaging during a designated period of time.

### 2.5. In Vivo Biodistribution and Tumor Imaging

In vivo NIR fluorescence imaging experiments were carried out using an FOBI imaging system (NeoScience, Suwon, South Korea). Mice injected with HA-COL-ZW (N = 3 independent experiments) were sacrificed at 24, 48, or 96 h post-injection. The fluorescence intensities of organs and tumor tissues collected from the mice, including heart, lungs, liver, pancreas, spleen, kidneys, duodenum, and intestine, were confirmed to analyze the time-dependent biodistribution of HA-COL-ZW. The obtained fluorescence images were analyzed by the open-source ImageJ software (National Institutes of Health, Bethesda, MD, USA).

### 2.6. In Vivo Photothermal Therapeutic Efficacy

The NCI-H460 tumor-bearing mice injected intravenously with HA-COL-ZW or PBS alone were anaesthetized after 24 h for PTT treatment, respectively. The tumor area was irradiated using an 808 nm laser with power density of 1.0 W/cm^2^ for 5 min. The mice were monitored using a thermal imager (FLIR Systems, Wilsonville, OR, USA) to confirm the temperature changes at tumor regions in real time. To evaluate the PTT efficacy, the tumor tissues were collected from the mice treated with PTT after 24 h of irradiation. The obtained tumor tissues were stained with hematoxylin and eosin (H&E) for histological observation. Additionally, the tumor growth in each treatment group was continuously observed up to 9 days to reconfirm the PTT efficacy. The measurement of tumor volume (V) in each treatment group was based on the following formula: V = 0.5 × longest diameter × (shortest diameter)^2^.

### 2.7. Statistical Analysis

Statistical analysis was carried out using one-way analysis of variance for multiple comparison test. A *p*-value less than 0.05 (typically *p* < 0.05) is statistically significant. The data were expressed as mean ± standard deviation (S.D.).

### 2.8. Histological Analysis

Tumor tissues harvested from each group were used for H&E staining and microscopic analysis. The tumor tissues were preserved in a deep freezer after fixation of the tumors using paraformaldehyde solution 4% in PBS. Subsequently, the 10 μm-thick cryosections of tumor samples were stained with H&E for microscopic analysis. Histological observation was carried out using a Nikon Eclipse Ti-U inverted microscope system (Nikon).

## 3. Results and Discussion

### 3.1. Preparation of HA-ZW, COL-ZW, and HA-COL-ZW

**Figure 1:**
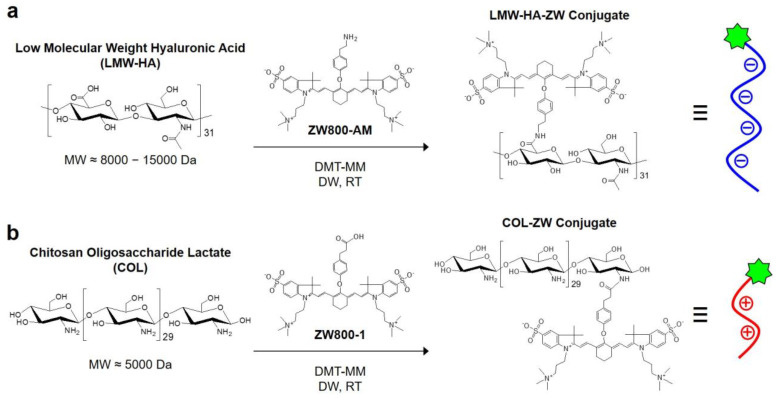
Synthetic scheme of (**a**) LMW-HA-ZW and (**b**) COL-ZW conjugates for NIR fluorescence imaging.

Experimental procedures for chemical synthesis of HA-ZW and COL-ZW conjugates are displayed in Figure 1. To prepare the HA-ZW conjugate, the carboxyl groups of LMW-HA were covalently conjugated with the amine-functionalized zwitterionic NIR dye (ZW800-AM) in the presence of a coupling agent, DMT-MM, to form amide bonds (Figure 1a). Firstly, the carboxyl group of LMW-HA can be activated by the DMT-MM; then, the electrophilic active ester of LMW-HA can undergo a nucleophilic substitution reaction with the amine group of ZW800-AM. Finally, the HA-ZW conjugate can be prepared via the formation of amide bonds between LMW-HA and ZW800-AM [30]. For the preparation of COL-ZW, the carboxyl-functionalized zwitterionic NIR dye (ZW800-1) was also conjugated to the amine groups of COL under the same condition (Figure 1b). To preserve the physicochemical properties of LMW-HA and COL, the conjugation reaction was conducted with a 1:1 molar ratio of LMW-HA to ZW800-AM or COL to ZW800-1. Moreover, the charge-balanced zwitterionic NIR dyes, such as ZW800-AM and ZW800-1, were chemically optimized for labelling peptides, especially small molecules, as demonstrated previously [31,32,33].

**Figure 2:**
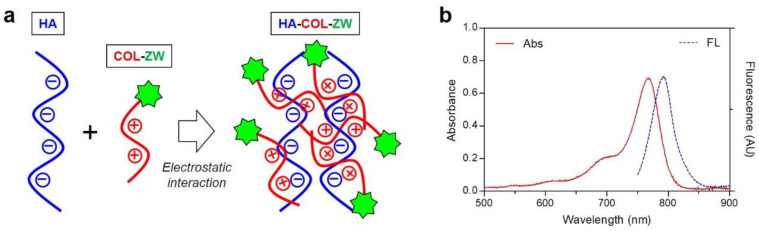
(**a**) Hypothetical scheme of the HA-COL-ZW nanocomplex. The nanocomplex formation of electrostatic interactions between negatively charged HA and positively charged COL-ZW. (**b**) Absorption and fluorescence emission spectra of HA-COL-ZW measured in PBS, pH 7.4.

As shown in Figure 2a, self-assembled nanocomplexes using anionic HA and cationic COL-ZW can spontaneously be formed by electrostatic interactions between the opposite charges. Moreover, the hydrogen bonds and van der Waals forces between anionic HA and cationic COL-ZW are also involved in the formation of stable nanocomplexes. In addition, the physicochemical properties of the self-assembled nanocomplexes can be varied by the charge ratio of the anionic-to-cationic species [34,35]. In this study, two kinds of HA-COL nanocomplexes in terms of mass ratios of LMW-HA and COL (1:1 and 1:2) were prepared to investigate their size and surface charge. The HA-COL-ZW nanocomplex formed by the 1:1 molar ratio of LMW-HA and COL displayed an absorption peak at 768 nm and fluorescence emission maximum at 792 nm, respectively (Figure 2b). The optical property of the HA-COL-ZW nanocomplex is suitable for in vivo NIR fluorescence imaging and further PTT applications after combining with the NIR laser irradiation system.

### 3.2. Size and Surface Charge Characterization of LMW-HA, COL, and HA-COL

**Figure 3:**
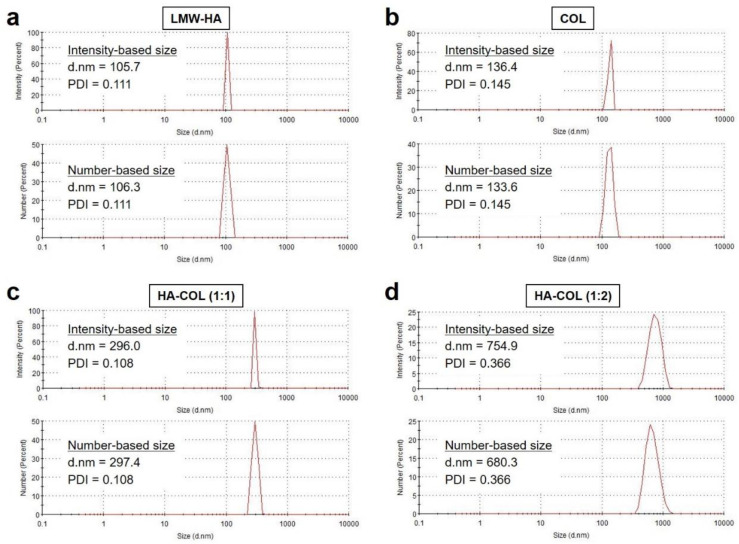
Size distributions of (**a**) LMW-HA, (**b**) COL, (**c**) HA-COL (1:1), and (**d**) HA-COL (1:2) before and after formation of nanocomplexes. The HA-COL nanocomplexes were prepared at varying molar ratios from 1:1 to 1:2 (LMW-HA:COL).

For DLS analysis, the size and surface zeta potentials for LMW-HA, COL, and HA-COL were measured at a concentration of 1 μM in DW. According to the guideline of DLS measurement, it is hard to measure the colored or fluorescing samples because laser light cannot be absorbed in colored solutions. Thus, the DLS analysis of LMW-HA, COL, and HA-COL was conducted without the NIR fluorescent dyes. The sizes of LMW-HA and COL were measured as 105.7 and 136.4 nm, respectively (Figure 3a,b). As expected, the size of HA-COL (1:1) was obtained as 296 nm, suggesting that the electrostatic interactions between anionic LMW-HA and cationic COL result in an increase in the particle size of HA-COL (Figure 3c). However, a significant increase in the size of the HA-COL nanocomplex was found as 754.9 nm when the HA-COL nanocomplex was prepared by the 1:2 molar ratio of LMW-HA and COL (Figure 3d). In this regard, it could be expected that the surface charge of HA-COL nanocomplexes would be more positive at higher molar ratios of COL. This is evident from the increased positive zeta potential values for higher chitosan concentrations, which is consistent with previous studies [36,37].

**Figure 4:**
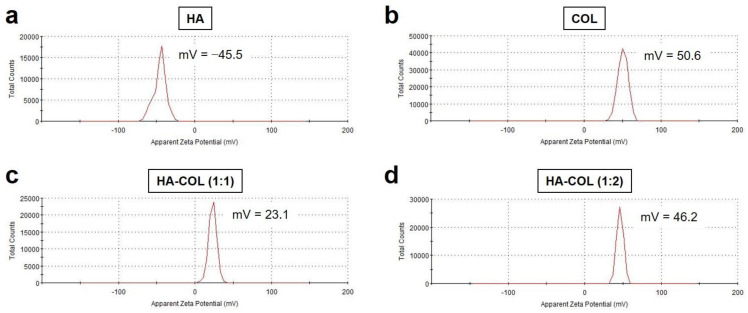
Zeta potential of (**a**) LMW-HA, (**b**) COL, (**c**) HA-COL (1:1), and (**d**) HA-COL (1:2) before and after formation of nanocomplexes. The HA-COL nanocomplexes were prepared at varying molar ratios from 1:1 to 1:2 (LMW-HA:COL).

As expected, zeta potential values of −45.5 and 50.6 mV were obtained for anionic LMW-HA and cationic COL, respectively (Figure 4a,b). Interestingly, the surface zeta potential of HA-COL (1:1) was found to be 23.1 mV, suggesting that the positive charge of COL is decreased by LMW-HA attachment (Figure 4c). However, HA-COL (1:2) recovered the surface charge towards positive zeta potential (46.2 mV) (Figure 4d). This indicates that the optimized formulation of the HA-COL nanocomplex can be determined to be a 1:1 molar ratio of anionic LMW-HA and cationic COL, considering an increase in the particle size of HA-COL (1:2). Since the positively charged chitosan-based nanoparticles offer several advantages such as enhanced cellular uptake [38], the HA-COL nanocomplex formed by a lower molar ratio of COL would result in a decrease in the surface charge.

### 3.3. In Vitro Cancer Cell Binding of HA-ZW, COL-ZW, and HA-COL-ZW

**Figure 5:**
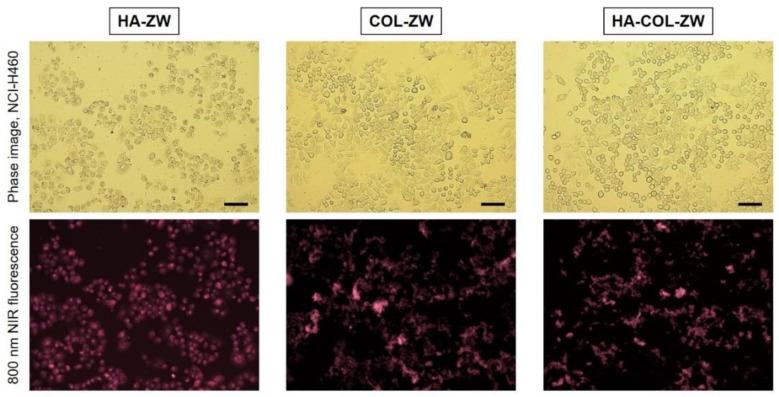
Live cancer cell binding of HA-ZW, COL-ZW, and HA-COL-ZW using the NCI-H460 cell line. In vitro NIR fluorescence imaging is conducted after treatment with 2 μM of each sample. The sample concentrations are determined by the concentrations of the ZW800-AM and ZW800-1 NIR dyes. Images are representative of N = 3 independent experiments. All NIR fluorescence images have identical exposure times and normalization. Scale bars = 100 μm.

To investigate the binding affinities of HA-ZW, COL-ZW, and HA-COL-ZW to cancer cells, 2 μM of each sample was incubated with the NCI-H460 human cancer cell line for 24 h at 37 °C, which was observed with an NIR fluorescent microscope after washing with PBS three times. The sample concentrations are determined by the concentrations of the ZW800-AM (*ε* = 184,000 M^−1^ cm^−1^) conjugated to HA-ZW, and ZW800-1 (*ε* = 246,000 M^−1^ cm^−1^) conjugated to COL-ZW and HA-COL-ZW, respectively [29]. As expected, HA-ZW can be specifically bound to CD44 receptors followed by internalization into the NCI-H460 cancer cells (Figure 5). It is well known that the CD44 was identified as a major cell surface receptor for HA, a component of the extracellular matrix, which is extensively overexpressed in NCI-H460 cancer cells [15,16]. Moreover, COL-ZW could also be localized to the cell boundaries with high fluorescence in the NCI-H460 cancer cells. This demonstrates that the cationic COL-ZW can adhere to the negatively charged outer surfaces of cell membranes, which is consistent with our previous study showing the binding specificity of COL-ZW in HT-29, MCF-7, and MDA-MB-231 human cancer cell lines [14]. Interestingly, HA-COL-ZW revealed similar binding patterns compared with those of COL-ZW in the NCI-H460 cancer cells. This indicates that the cationic surface charge of HA-COL-ZW can play a more important role in the cellular uptake of HA-COL-ZW rather than the HA component binding to the CD44 receptor.

### 3.4. Time-Dependent In Vivo Tumor Imaging and Biodistribution

**Figure 6:**
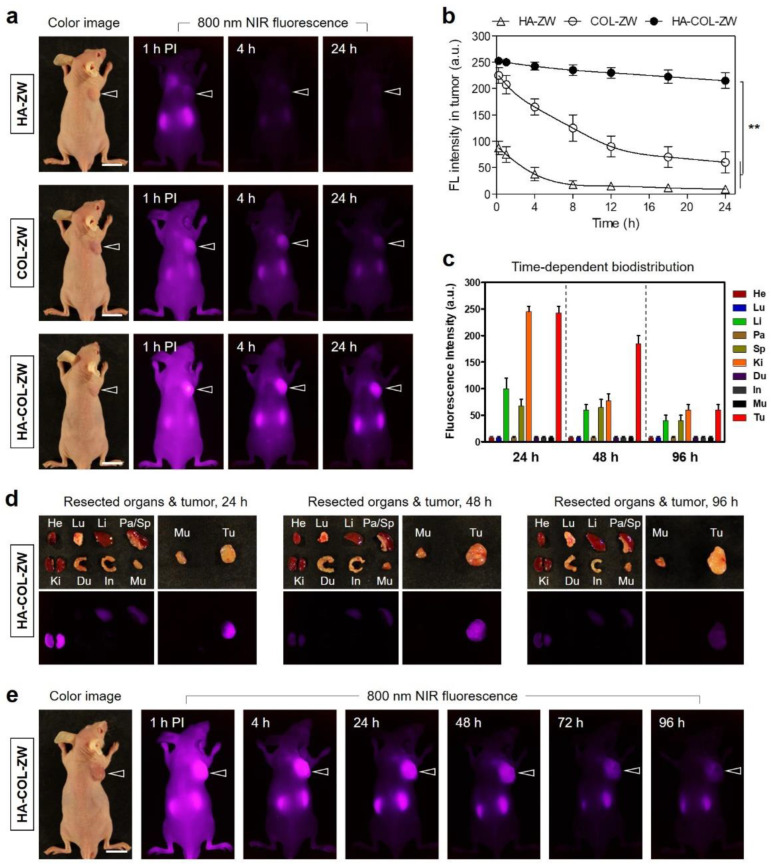
In vivo NCI-H460 tumor targeting efficiency and biodistribution of HA-ZW, COL-ZW, and HA-COL-ZW. (**a**) NIR fluorescence imaging 24 h after injection of HA-ZW, COL-ZW, and HA-COL-ZW, respectively. (**b**) Time-dependent fluorescence intensities at the tumor sites targeted by HA-ZW, COL-ZW, and HA-COL-ZW. (**c**) Quantitative fluorescence analysis of intraoperative dissected organs at 24 h, 48 h, and 96 h post-injection of HA-COL-ZW. (**d**) Resected organs and tumors imaged at 24 h, 48 h, and 96 h post-injection of HA-COL-ZW. (**e**) NIR fluorescence imaging for 96 h post-injection of HA-COL-ZW. Images are representative of 3 mice per treatment group (N = 3 independent experiments). The tumor tissue is pointed with arrowheads. Abbreviations: Du, duodenum; He, heart; In, intestine; Ki, kidneys; Li, liver; Lu, lungs; Mu, muscle; Pa, pancreas; Sp, spleen; Tu, tumor; and PI, post-injection. Data are expressed as mean ± S.D. (*n* = 3, ** *p* < 0.01). Scale bars = 1 cm.

After determining the cellular uptake behavior of HA-ZW, COL-ZW, and HA-COL-ZW, the in vivo accumulation of HA-ZW, COL-ZW, and HA-COL-ZW in the tumor was subsequently investigated in the NCI-H460 xenograft tumor model. The three samples diluted in PBS (10 nmol/100 μL based on the NIR dye concentration) were intravenously injected into the tumor-bearing mice. Then, the mice were monitored in real time at different time points for 24 h (Figure 6a). Unexpectedly, the tumor injected with HA-ZW showed no significant uptake within 4 h after injection, and the HA-ZW was rapidly excreted from the body at 24 h post-injection. This result indicates that the molecular weight and rapid body clearance of HA played a critical role in tumor accumulation. It is also reported that the HA receptor for endocytosis (HARE) shows size-dependent binding affinity with HA. It can bind with HA in a molecular weight ranging from 40 to 400 kDa, while smaller and larger HA was inactive. The optimum HARE signaling size was ~140 kDa [39,40]. As expected, tumors exhibited good uptake of COL-ZW for up to 4 h after injection, which is consistent with our previous study [14]. Remarkably, the tumor-bearing mice injected with HA-COL-ZW revealed high accumulation in the tumor and the fluorescence intensity was maintained consistently until 24 h post-injection (Figure 6b). This result demonstrates that the increased size and decreased surface charge of COL-ZW after combining with LMW-HA contribute to the enhanced tumor accumulation and prolonged blood circulation of HA-COL-ZW compared with that of COL-ZW. Moreover, we further confirmed the biodistribution and clearance of HA-COL-ZW until 96 h after injection (Figure 6c). Interestingly, HA-COL-ZW showed low nonspecific uptake in the body 24 h post-injection. Based on the fluorescence intensities of resected organs and tumors, the HA-COL-ZW nanocomplex was highly accumulated in the tumor and mostly cleared into the kidneys as a result of improved biodistribution and clearance in the body (Figure 6d). Eventually, the HA-COL-ZW nanocomplex was cleared out at 96 h after injection without nonspecific uptake in other tissues/organs. The optimal time for PTT treatment to avoid unnecessary damage to adjacent normal tissues was determined at 24 h after injection of HA-COL-ZW. (Figure 6e). The results demonstrate that the LMW-HA played a key role in enhanced tumor accumulation with prolonged tumor retention of HA-COL-ZW and may provide insights for designing the optimal LMW-HA/COL nanocomplexes to maximize the therapeutic efficacy of tumor-targeted photothermal therapy.

### 3.5. In Vivo Photothermal Therapeutic Efficacy

**Figure 7:**
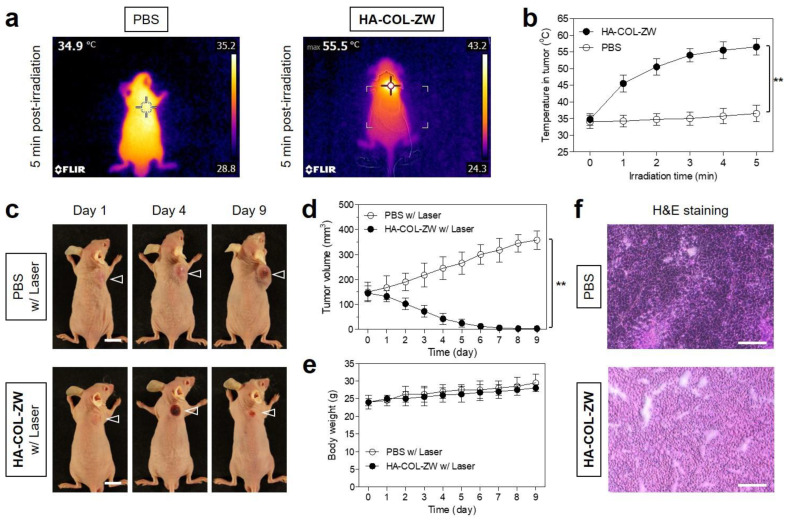
(**a**) Thermal images of and (**b**) temperature changes in tumor-bearing mice at the tumor area 24 h after injection of PBS or HA-COL-ZW, followed by 808 nm laser irradiation (1.0 W/cm^2^) for 5 min. (**c**) Photos of tumor size changes in each treatment group observed for 9 days. The tumor area is pointed with arrowheads. Scale bars = 1 cm. Photos are representative of each treatment group. (**d**) Tumor growth and (**e**) body weight change in each treatment group were observed for 9 days. (**f**) Histological observation of tumors stained with H&E in each treatment group. Scale bars = 100 μm. Data are expressed as mean ± S.D. (*n* = 3, ** *p* < 0.01).

To further confirm the NIR laser-induced PTT effect of the HA-COL-ZW nanocomplex, an in vivo PTT capability study using NCI-H460 tumor-bearing mice was conducted in different groups (three mice per treatment group), treated with PBS alone and HA-COL-ZW under NIR laser irradiation. The tumor-bearing mice were treated with HA-COL-ZW (10 nmol/100 μL based on the ZW800-1 concentration), followed by 808 nm NIR laser irradiation at 1.0 W/cm^2^ for 5 min at 24 h post-injection. Interestingly, the power density of the 808 nm NIR laser could be reduced in this study compared with that of previous studies (1.1 W/cm^2^), which is the power density typically used in our group, owing to the highly enhanced tumor accumulation of HA-COL-ZW [14]. As shown in Figure 7a, the tumor temperature in the HA-COL-ZW treatment group rapidly increased up to 55.5 °C during the 5 min of laser irradiation, whereas the temperature of the tumors treated with PBS alone showed little change (34.9 °C) under the same condition. This demonstrates that the HA-COL-ZW nanocomplex accumulated in the tumor at 24 h after injection was sufficient to produce photothermal energy for complete tumor ablation. The temperature of the tumors treated with HA-COL-ZW immediately reached ~50 °C within the first 2 min after irradiation, which is a phototherapeutic temperature high enough to kill cancer cells, and maintained up to ~55 °C in the remaining 3 min of laser irradiation, showing excellent PTT capability in vivo (Figure 7b). On the contrary, the PBS-treated group displayed no significant change in the temperature in tumors during laser irradiation, suggesting that the combination of the HA-COL-ZW nanocomplex and NIR laser induced the photothermal effect.

The tumor sizes of mice in two groups were continuously monitored and measured for 9 days after PTT treatment to evaluate the phototherapeutic efficacy (Figure 7c,d). As expected, the tumor volumes in the PBS group increased gradually for 9 days without tumor suppression induced by laser irradiation alone, whereas the tumors in the HA-COL-ZW treatment group showed a significant PTT effect with complete tumor ablation and no recurrence for 9 days after laser irradiation. Thus, this result indicates that the tumor targetability of PTT agents is highly important for safe and accurate PTT because the treatment with laser irradiation alone did not cause the phototherapeutic effect as well as normal tissue damage. Further, the body weight change in mice was observed to investigate the systemic toxicity of HA-COL-ZW. As a result, the mice in each treatment group revealed no significant loss of body weight during the treatment period. This demonstrates that the HA-COL-ZW nanocomplex is a safe and biocompatible PTT agent (Figure 7e). Furthermore, the tumor tissues were harvested from each group after 24 h of different treatments and used for histological examination via H&E staining. Importantly, the tumor structure in the PBS and laser treatment group showed no damage, while complete necrosis was observed after laser irradiation in the HA-COL-ZW treatment group (Figure 7f). This demonstrates that the HA-COL-ZW nanocomplex can be successfully used as a biocompatible phototheranostic agent for safe and effective in vivo PTT application.

## 4. Conclusions

In summary, a new type of LMW-HA/COL-based phototheranostic nanocomplex, HA-COL-ZW, was rationally prepared and utilized for fluorescence-guided photothermal cancer therapy. In this study, two different natural polymers were used simultaneously to improve the tumor-specific accumulation with prolonged tumor retention: LMW-HA for helping to increase the tumor uptake and retention of COL with charge compensation, and COL for playing the leading role in tumor targeting with high affinity. Furthermore, the ZW800-1 that conjugated to COL could not only act as a fluorescence imaging agent but also show efficient photothermal energy conversion under NIR laser irradiation, thereby resulting in complete tumor ablation with no incidence of recurrence. Therefore, the bifunctional HA-COL-ZW nanocomplex developed here has great potential as a biocompatible theranostic agent for tumor-targeted imaging and phototherapy.

## Data Availability

Not applicable.
